# Photothermally active nanoparticles as a promising tool for eliminating bacteria and biofilms

**DOI:** 10.3762/bjnano.11.98

**Published:** 2020-07-31

**Authors:** Mykola Borzenkov, Piersandro Pallavicini, Angelo Taglietti, Laura D’Alfonso, Maddalena Collini, Giuseppe Chirico

**Affiliations:** 1 Department of Medicine and Surgery, Nanomedicine Center, University of Milano-Bicocca, piazza dell’Ateneo Nuovo, 20126, Milan, Italy; 2 Department of Chemistry, University of Pavia, via Taramelli 12, 27100, Pavia, Italy; 3 Department of Physics, University of Milano-Bicocca, piazza dell’Ateneo Nuovo, 20126, Milan, Italy

**Keywords:** antibacterial activity, bacteria eradication, nanoparticles, NIR light, photothermal effect

## Abstract

Bacterial contamination is a severe issue that affects medical devices, hospital tools and surfaces. When microorganisms adhere to a surface (e.g., medical devices or implants) they can develop into a biofilm, thereby becoming more resistant to conventional biocides and disinfectants. Nanoparticles can be used as an antibacterial agent in medical instruments or as a protective coating in implantable devices. In particular, attention is being drawn to photothermally active nanoparticles that are capable of converting absorbed light into heat. These nanoparticles can efficiently eradicate bacteria and biofilms upon light activation (predominantly near the infrared to near-infrared spectral region) due a rapid and pronounced local temperature increase. By using this approach new, protective, antibacterial surfaces and materials can be developed that can be remotely activated on demand. In this review, we summarize the state-of-the art regarding the application of various photothermally active nanoparticles and their corresponding nanocomposites for the light-triggered eradication of bacteria and biofilms.

## Introduction

Bacteria are considered the major source of hospital-acquired nosocomial infections and patients are at a risk higher than 13.5% of contracting these diseases [1–2]. In addition, the bacterial strains are becoming increasingly resistant to conventional drug treatments [3]. One reason for bacterial resistance is that after attaching to a surface, bacteria can generate biofilms. Biofilms are organized sessile microbial aggregates protected by self-generated extracellular polymeric substances [1,4]. Biofilms are resistant to mechanical abrasion and drug treatments, including antibiotics. The detachment of a single bacterial cell or biofilm fragments can result in systemic chronic infections [5]. As a consequence, when a biofilm forms at the surface of prostheses, catheters or other implantable devices, surgical removal is the only possible solution to prevent the infection from spreading [6]. Aside from the hospital environment, the bacterial contamination and the biofilm formation also affect the food packaging, textile and water treatment industries.

As the conventional drugs and agents fail to eradicate bacteria and biofilms, the search for new tools has become a hot research topic worldwide. Several studies have reported that various types of nanomaterials (both inorganic and organic) have demonstrated promising results regarding antibacterial activity. The advances in the field of nanomaterials exhibiting antibacterial activity are well summarized in recent reviews [1,7–9]. In particular, the antibacterial properties of silver nanoparticles (Ag NPs) and Ag-NP-based polymeric materials are the most intensively studied within the broad spectrum of existing nanomaterials [10–15]. However, the low stability (even at physiological pH levels) and the potential toxicity of Ag NPs limit their application [7,16]. Moreover, the results regarding the efficiency of Ag NPs in preventing biofilm formation are controversial as the Ag nanoparticles seem to be effective against the Gram-negative but not so much against the Gram-positive strains [6]. The antibacterial properties of a wide range of nanoparticles and their corresponding nanocomposites, such as ZnO [17–18], TiO_2_ [19–20], Fe_3_O_4_ [21–23] and CuO [24–26], are also well described in the literature. The antibacterial activity of polymeric nanoparticles, such as the polystyrene sulfate coated with a bilayer of dioctadecyldimethylammonium bromide [27] and poly(lactic-*co*-glycolic acid) loaded with gentamicin [28], were also studied. The state-of-the art in antimicrobial polymeric nanoparticles, with an emphasis on the relationship between their structure and activity, is well presented in a recent review [29]. The antibacterial properties of solid lipid nanoparticles are also a subject of specific research interest as they have advantages over other NPs, such as controlled and sustained release, enhanced solubility and biocompatibility [30–32].

Within the wide variety of existing nanomaterials with antibacterial properties, photothermally active nanoparticles, with absorption in the visible–near-infrared (NIR) region, are receiving great attention as they are can, upon irradiation, increase the local temperature in the surrounding medium and consequently thermally inactivate different types of bacteria [2,33]. This approach can provide an alternative and direct in situ sterilization as opposed to the conventional sterilization procedures that are usually time consuming and require higher temperatures. In addition, the NIR light in the so-called “bio-transparent window” (750–900 nm) is considered safe for direct in vivo application as it has a large penetration depth and does not damage normal tissue (at least if used under given irradiance limits, e.g., 0.32 W/cm^2^ at 800 nm) [2,34]. Moreover, stable and reproducible photothermal properties will bring additional advantages for efficient in situ sterilization.

Therefore, in this review we focus on the application of photothermally active nanoparticles, nanoparticle-based surfaces and nanocomposite materials to bacteria and biofilm ablation. This is a relatively recent approach and that could be applied as a promising alternative to existing antibacterial treatments. The state-of-the art and future perspectives are highlighted here.

## Review

### Photothermally active nanoparticles: a brief overview

Nowadays, there is a broad spectrum of nanoparticles that are able to convert absorbed light into heat through a phenomenon known as the photothermal effect [33,35]. These nanoparticles are predominantly inorganic, constituted by noble metals (Au, Ag), carbon-based materials, nanoscale metal chalcogenides (Cu_2−_*_x_*E, E = S, Se, Te), transition metal dichalcogenide nanostructures (e.g., WS_2_, MoS_2_), metal-oxide nanoparticles (e.g., WO_3_), and nanoscale coordination compounds (e.g., Prussian blue nanoparticles) [33,36–38]. The photothermal properties of these nanoparticles are due to the resonant oscillation of the surface electrons, called surface plasmons (e.g., plasmonic gold and silver nanoparticles) [38], or they are due to the energy of the band transitions (e.g., Cu^2+^ *d*–*d* transition in CuS nanoparticles) [39]. Under visible–NIR light irradiation, these nanoparticles produce thermal relaxation, leading to a local increase in the temperature. The overall photothermal effect depends on the irradiation intensity and wavelength, nanoparticle concentration, and the nanoparticle photothermal conversion efficiency [33,38]. It also depends on the type of the nanomaterial used, for example, aqueous solutions, self-assembled monolayers or polymeric nanocomposites [33]. In the case of plasmonic nanoparticles, the plasmonic properties (i.e., the maximum position of the absorption band and the band shape) are strongly affected by the conditions under which the nanoparticles are synthesized, resulting in nanoparticles with different dimensions and shape. On the other hand, the nanoparticle surface chemistry as well as the surrounding environment exert a weaker influence on the plasmonic properties. For the non-plasmonic nanoparticles, the photothermal properties are less dependent on their size, shape and surrounding environment [35,40].

**Figure 1:**
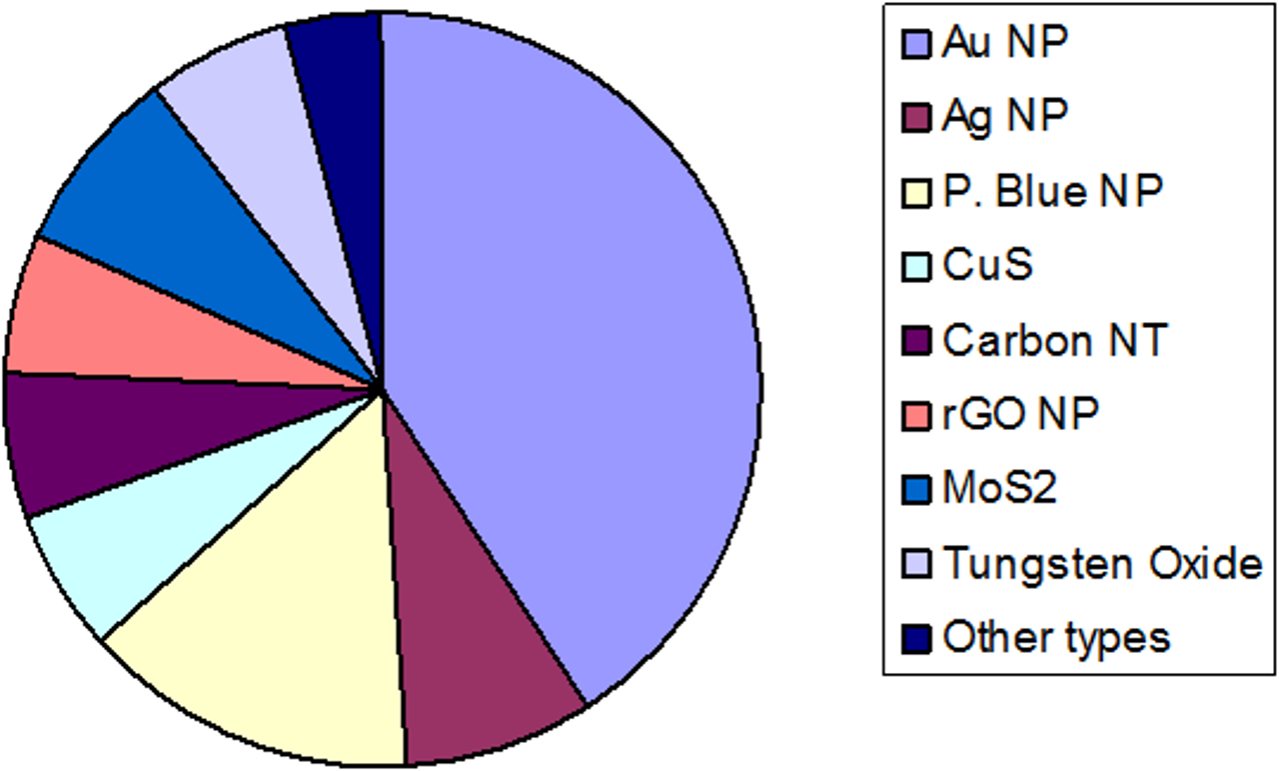
Pie chart showing the ratio of photothermally active nanoparticles (NPs) used for the temperature-induced bacteria and biofilm ablation.

**Figure 2:**
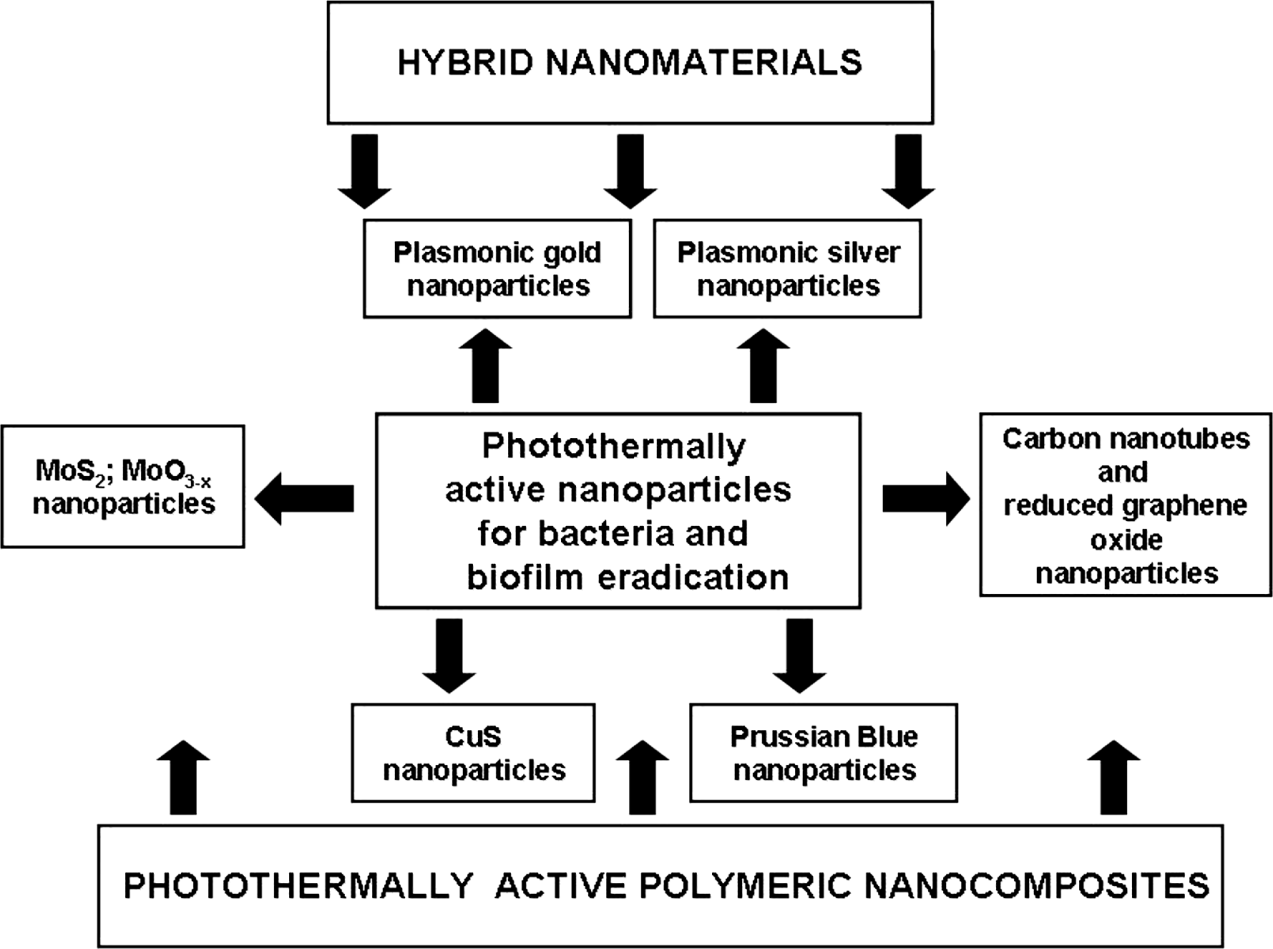
Schematic showing the relationship between photothermally active nanoparticles and nanocomposites for bacteria and biofilm eradication.

The photothermally active nanoparticles have vast potential for application in nanomedicine and biotechnology. The most important examples of their application are: hyperthermic cancer cell ablation and photothermally induced drug release [41–42], in addition to new quantitative tools for biochemical analysis [43] and photothermally induced cell stimulation [44–45]. Since bacteria and biofilm photothermal ablation is another promising application that is currently being widely investigated, this review will highlight the current progress made in the field due to the use of photothermally active nanoparticles and nanocomposites. For clarity, we decided to focus on the antibacterial effect triggered by the temperature increase upon light irradiation either as a direct action or as a combination of synergic effects. The photothermally active antibacterial nanoparticles found in the literature are shown in Figure 1. The outline of this review is summarized in Figure 2.

### Plasmonic gold nanoparticles for bacteria and biofilm photothermal ablation

According to our literature search, gold nanoparticles in various dimensions and shapes are the most widely studied nanomaterial for the photothermal ablation of bacteria and biofilms. This may be explained by the fact that some of these gold nanoparticles are commercially available and are also widely applied in other fields of nanotechnology and nanomedicine. Therefore, the chemistry involved in their preparation as well as their properties and stability are well studied. In addition to temperature-induced effects, photothermal ablation may induce other phenomena such as the generation of reactive oxygen species [46], which can increase the antibacterial action. One pioneering work in this field demonstrated the selective elimination of bacteria targeted with photothermally active gold nanoparticles conjugated with specific antibodies [47]. The strong laser-induced overheating effects accompanied by the bubble-formation phenomena around the clustered gold nanoparticles were the main causes of bacterial damage. The polygonally shaped vancomycin-bound gold nanoparticles efficiently killed (>99%) of the targeted bacteria that were submitted to irradiation with 808 nm light for 5 min [48]. In another study, gold nanorods covalently linked with primary antibodies efficiently eliminated the pathogenic *P. aeruginosa* upon NIR irradiation [49]. Gold nanorods were also applied to efficiently ablate (up to 97%) *S. aureus* upon NIR laser irradiation [50]. In these studies, the results suggested that the thermal damage of the bacterial membranes led to bacterial death.

In 2013, the first examples in which the photothermal effect was applied on gold nanomaterials (nanorods, nanoshells, silk hydrogel containing spherical gold nanoparticles) to induce antibacterial activity were summarized in a review [51]. Later on, in 2017, another review highlighted the advances in the gold nanoparticle field regarding the use of several gold nanostructures, such as nanocages, nanorods, nanostars, and core–shell silver/gold nanoparticles for laser-driven hyperthermal ablation of multi-drug resistant bacteria [52]. Therefore, we will focus here on the latest achievements in this field including the application of gold-nanoparticle-coated surfaces and nanocomposite materials for antibacterial treatments.

Gold nanoshells functionalized with carboxylate-terminated organosulfur ligands were attached to the surface of a model catheter and their effectiveness in eliminating adhering *E. faecalis* bacteria was tested [53]. It was shown that gold-nanoshell-modified surfaces can effectively kill *E. faecalis* on silicone surfaces under NIR irradiation. Several bacterial strains (*E. coli*, *Bacillus subtilis*, *Exiguobacterium sp.* AT1b), deposited on surfaces precoated with nanoporous gold disks, were inactivated after a short photothermal NIR light exposure (8.5 W/cm^2^) [2]. The authors showed that, at the irradiation spot, the temperature rose above 200 °C within 25 s, which was enough to completely ablate the pathogenic bacteria.

In a recent publication, a novel theranostic strategy based on bacteria-induced gold nanoparticle aggregation was implemented [54]. According to this strategy, the spherical nanoparticles, with a typical localized surface plasmon resonance (LSPR) absorption at 520 nm, aggregated in situ at the surface of the bacterial membrane. The gold nanoparticle aggregation induced a change in the observed LSPR band, increasing both the NIR absorption and the photothermal conversion, which allowed for the elimination of Gram-positive bacteria. In another recent work, the photothermal-triggered effect of phospholipid-coated gold nanorods against *P. aeruginosa* planktonic and biofilm cultures was studied [55]. The authors found that the interaction between a planktonic culture of *P. aeruginosa* and a nanoparticle suspension, under NIR light exposure, resulted in a ≈6 log cycle reduction in the bacterial viable count with respect to the control. The percentage reduction in the *P. aeruginosa* biofilm viable count was ≈2.5–6.0 log cycle upon laser excitation with different gold nanoparticle concentrations. Previously, the same research group studied the photothermal bactericidal activity of hydrophilic and hydrophobic functionalized gold nanorods against *S. aureus* and *Propionibacterium acnes* by measuring the percentage reduction in viable bacteria upon NIR light excitation [56]. The local temperature increase showed a significant photothermal ablation effect (≥99.99%) on these strains.

Surface-adaptive 14 nm gold nanoparticles exhibiting multiple functions, such as self-adaptive targeting to the acidic biofilm microenvironment and an enhanced photothermal ablation of methicillin-resistant *S. aureus* biofilm under NIR light irradiation (laser intensity 0.91 W/cm^2^), were previously reported [57]. The gold nanoparticles were modified by pH-responsive mixed-charged zwitterionic self-assembled monolayers consisting of a weak electrolytic (i.e., pH-sensitive), 11-mercaptoundecanoic acid, a strong electrolytic (i.e., pH-independent), and (10-mercaptodecyl)trimethylammonium bromide. In addition, the resulting photothermal effect did not cause damage to the surrounding healthy tissue.

Multibranched gold nanoparticles (nanocrosses) with an efficient absorption in the NIR region and a fast photothermal response under NIR light excitation were conjugated to secondary and primary antibodies against PcrV, a type III secretion protein that is expressed by the superbug bacteria *P. aeruginosa* [58]. It was demonstrated that these conjugated nanoparticles completely obliterated *P. aeruginosa* and its biofilms upon NIR irradiation for 5 min with an 800 nm laser at a low intensity (3 W/cm^2^). Notably, no bacterial activity was detected 48 h after the procedure, confirming that the heat generated from the irradiated gold nanocrosses attached to the bacteria was effective in eliminating and preventing bacterial regrowth.

By combining the magnetic and optical properties of Fe_3_O_4_ and gold nanoparticles, respectively, multifunctional nanohybrids based on Fe_3_O_4_@Au (i.e., magnetite nanoparticles decorated with gold nanoparticles) were developed to rapidly detect and inhibit pathogenic microorganisms [59]. By using the label-free surface-enhanced Raman spectroscopy technique, the authors demonstrated that the optical fingerprints can be used to sense bacterial cell molecular structures and to promote recyclable photothermal ablation of different bacterial strains (Gram-positive, Gram-negative, and anaerobic bacteria). A chemo-photothermal therapeutic hybrid based on polydopamine-coated gold nanorods was also developed [60]. Such coating acquired a high Ag^+^ loading efficiency. It was found that apart from the direct NIR-induced hyperthermal effect on the bacteria elimination, the hyperthermia could also trigger a release of more Ag^+^ ions as a synergic effect.

New methods for detecting and eliminating antibiotic-resistant, Gram-negative bacteria were developed by conjugating the phages to gold nanorods, whose excitation by near-infrared light at 808 nm causes localized heating (up to 81 °C) that is capable of destroying the bacteria nearby [61].

**Figure 3:**
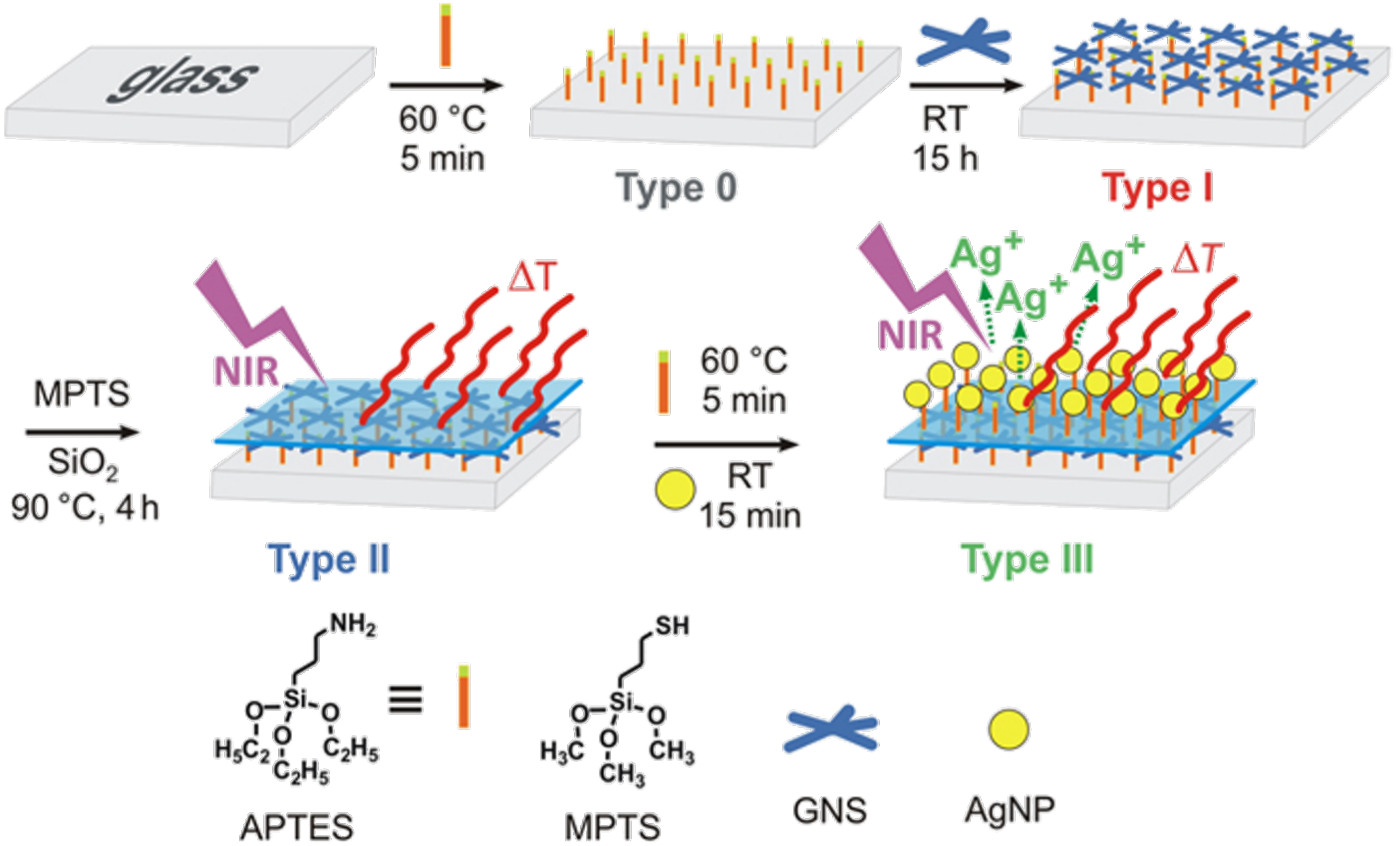
Preparation of a dual-function antibacterial surface with photo-switchable activity and sustained biocidal release. Figure reprinted with permission from [62], copyright 2017, under the terms of the Creative Commons CC BY License, http://creativecommons.org/licenses/by/4.0/.

Gold nanostar monolayers with a tunable LSPR absorption were grafted onto glass slides and were found to efficiently eliminate an *S. aureus* biofilm upon NIR laser excitation [6]. Later, these authors introduced a new photo-responsive antibacterial surface preparation approach by combining the on-demand photo-switchable hyperthermal action and the sustained biocidal Ag^+^ release from an upper layer of Ag NPs by sequentially grafting and segregating gold nanostars and Ag NPs, as shown in Figure 3 [62]. The proposed strategy simultaneously unraveled the static and photo-activated contributions to the overall antibacterial performance of the surfaces. In this case a pronounced synergistic effect between the photothermal action of the gold nanostars (applying intensities of 0.25 W/cm^2^ at 808 nm, lower than the ANSI limits) and the intrinsic nanochemical Ag NP bactericidal effect was observed.

In order to combat bacterial adhesion and proliferation, recent techniques have been developed to functionalize photothermally active monolayers of gold nanostars on glass with thiol monolayers, aiming to impart a different wettability to the surfaces [63]. It was verified that the photothermal features and the consequent hyperthermia-derived antibacterial effects were not affected by the thiol layers, which enable the eradication of at least 99.99% of the bacterial strains.

In 2018, an interesting approach demonstrated that a paper surface functionalized with gold nanoparticles conjugated with graphene oxide showed NIR laser-triggered photothermal ablation of pathogenic bacteria [64]. Upon NIR light exposure, the fabricated paper generated a temperature increase of over 80 °C, sufficient for the photothermal ablation of both Gram-positive (*Bacillus subtilis* and *S. aureus*) and Gram-negative bacteria (*E. coli* and *P. aeruginosa*).

### Polymeric nanocomposites containing photothermally active gold nanoparticles for bacteria and biofilm ablation

Despite the increasing number of successful studies, the direct application of colloidal solutions containing photothermally active gold nanoparticles may be limited particularly due to the risk of accumulation in the body. Moreover, due to the heat dissipation in aqueous solution, high laser intensities are usually required to achieve the desired temperatures, thus restricting the practical implementation of the bacteria photothermal eradication [33]. The gold nanoparticle self-assembly on glass surfaces is a generally time-consuming process with a relatively poor reproducibility. These restrictions may also limit the full implementation of the photothermally triggered bacterial ablation approach at the surface of indwelling devices. Instead, the embedding of photothermally active nanoparticles into polymeric matrices allows for the fabrication of uniform materials that can be remotely activated on demand with a high photothermal efficiency, while preserving the original photothermal properties of these nanoparticles [33,38]. This strategy may also offer a powerful alternative for the preparation of new antibacterial bulk materials and objects, and for film-coating the surface of preformed objects in any shape. The laser-induced heating of gold nanoparticles embedded in an injectable silk protein hydrogel was one of the first examples that used this approach [65]. The NIR laser irradiation at 528 nm for 15 min (450 mW) elevated the maximum gel temperature to 59 °C. The in vivo studies demonstrated a sufficient bacterial reduction after one round of laser exposure.

**Figure 4:**
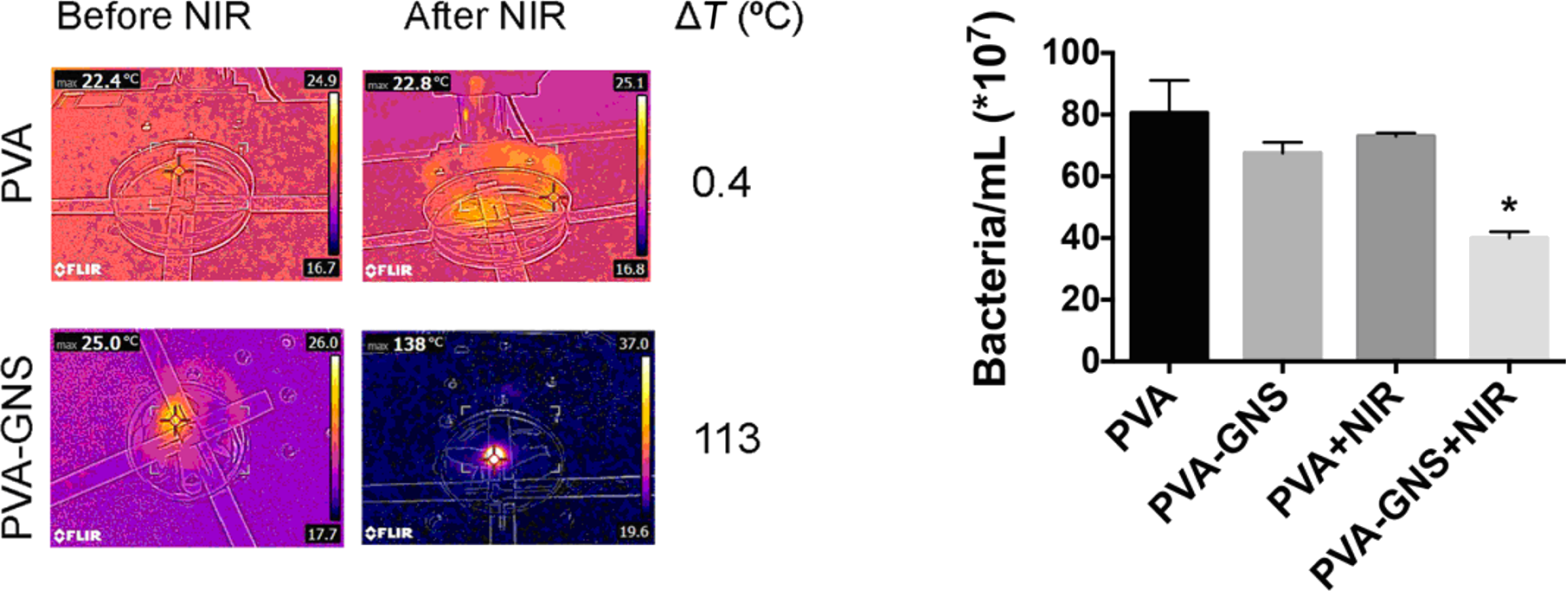
Left panel: temperature increase upon 1064 nm laser irradiation of bare PVA films and PVA films containing gold nanostars (GNSs). Right panel: Bacteria ablation on PVA/gold nanostar films as result of the NIR-induced photothermal effect. Figure reprinted with permission from [66], copyright 2018, under the terms of the Creative Commons Attribution License, http://creativecommons.org/licenses/by/4.0/.

Poly(vinyl alcohol) (PVA) films cross-linked with citric acid loaded with highly efficient photothermal gold nanostars were also fabricated [66]. The resulting films demonstrated a pronounced photothermal effect under NIR irradiation in the 730–1064 nm wavelength range. The local photothermal effect triggered by the NIR irradiation of PVA-GNS films was highly efficient in eliminating *E. coli* bacteria, as shown in Figure 4.

In a very recent study, the antimicrobial activity of a chitosan-based hydrogel with embedded gold nanorods under low-power (200 mW) diode laser irradiation was reported [67]. The antibacterial activity was measured on both Gram-positive and Gram-negative strains, including clinically isolated multidrug-resistant pathogens. The authors found that the fabricated nanocomposite showed pronounced antimicrobial activity against tested pathogenic microorganisms with limited cytotoxicity. In another recent publication, the photothermal effect of phospholipid-coated gold nanorods loaded into a poloxamer 407 hydrogel resulted in ≈4.5–5 log cycle reduction in the viable counts of a *P. aeruginosa* biofilm in comparison to the control sample [55]. The authors did not discern a difference in bacteria eradication with respect to the laser irradiation modality (continuous or pulsed).

A flexible patch capable of rapidly treating subcutaneous wound infections upon NIR light irradiation was fabricated [68]. This patch combined the photothermal properties of gold nanohole arrays with reduced graphene oxide nanosheets in a unique and flexible polyimide film for laser-gated pathogen inactivation. For the in vivo experiments, the patch was irradiated for 5 min with an LED array (940 nm, 10 W) and the patch surface temperature increased to 52 °C. These tests indicated that there was wound healing of the infected site, while untreated areas resulted in necrotic muscular fibers and bacterial infiltration.

### Applications of other types of nanoparticles for photothermally induced bacteria and biofilm ablation

Besides the widely studied gold nanoparticles, other inorganic nanoparticles exhibiting efficient photothermal properties have demonstrated promising results in the field of NIR-triggered bacterial eradication. An important advantage is that such nanoparticles are usually made from materials that are less expensive than gold and, in some cases, the time-sustained release of the antibacterial ions can act synergistically with the NIR-induced hyperthermia to eliminate the bacteria and biofilm.

**Figure 5:**
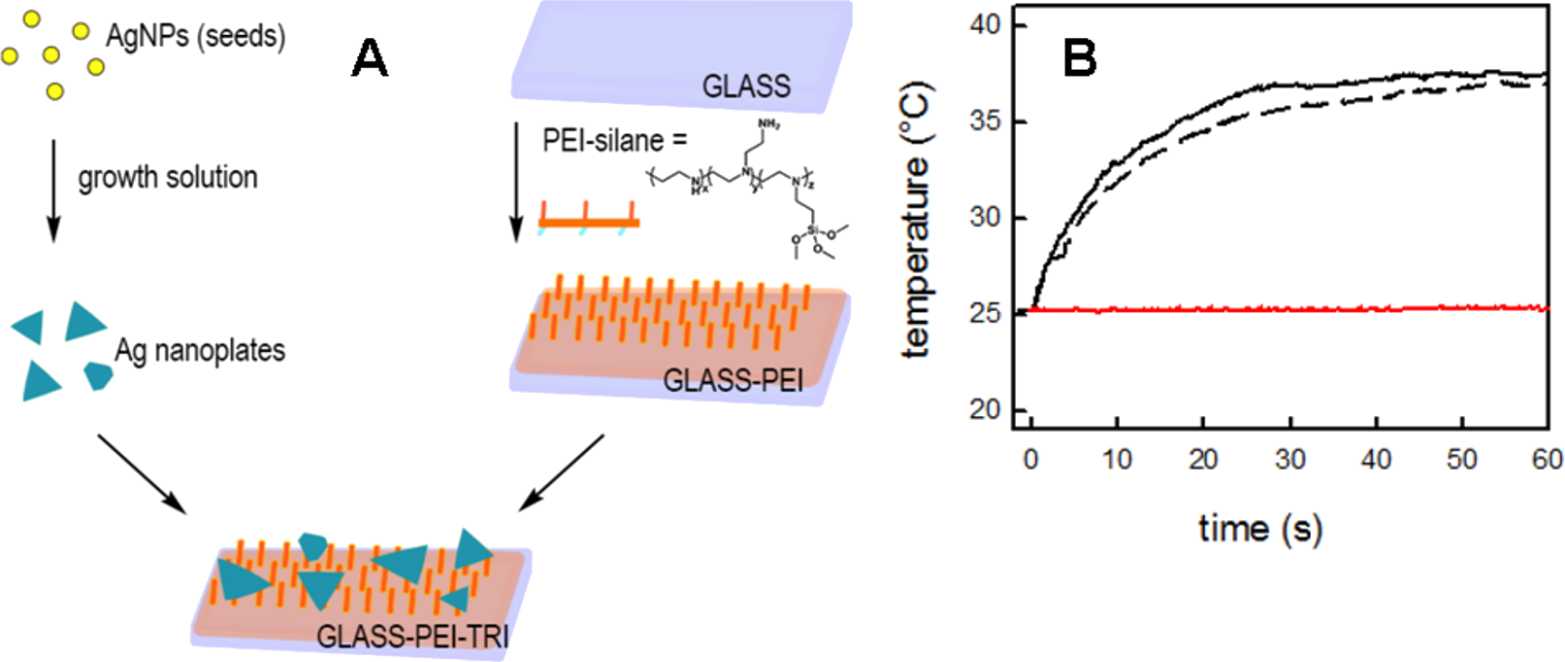
A synthetic preparation route for silver nanoplates on polyethylenimine (PEI)-functionalized glass (A). Temperature increase of grafted silver nanoplates under NIR irradiation (808 nm, 0.26 W/cm^2^). The red line corresponds to the NIR irradiation on a bare glass surface (B). Figure reprinted with permission from [71], copyright 2017, under the terms of the Creative Commons CC BY License, http://creativecommons.org/licenses/by/4.0/.

Silver nanoparticles that absorb in the NIR spectral range can be used for the photothermal elimination of bacteria and this physical effect can be enhanced by both the release of Ag^+^ and by the so-called nanomechanical effect (i.e., a disruptive phenomenon that is generated by the high NP surface energy at the bacterial membrane) [69]. A reproducible synthetic method was developed to grow anisotropic silver nanoplates on glass with a LSPR absorption tunable in the NIR range [70]. The fabricated materials demonstrated a strong antibacterial activity, which is based mainly on two different mechanisms: the silver ion release and photothermal-induced hyperthermia under NIR laser irradiation. Later, it was shown that similar silver nanoparticles (i.e., nanoplates) could be preassembled and grafted on a polyethylenimine (PEI)-functionalized glass surface, as depicted in Figure 5. Also such surfaces demonstrated both an intrinsic and a NIR-irradiation-enhanced antibacterial effect towards Gram-positive (*S. aureus*) and Gram-negative (*E. coli*) strains [71].

Silver core nanoparticles with a magnetic Fe_3_O_4_ shell, displaying strong magnetic responsiveness and tunable plasmonic properties (absorption maximum in the 480–825 nm range), were prepared as a multifunctional tool for bacterial disinfection [72]. The prepared nanoparticles displayed enhanced photothermal stability, high magnetic recyclability and low cytotoxicity. It was shown that even at low concentration (25 ppm) the nanoparticles could kill 100% of the *E. coli* (10^7^ CFU mL^−1^) within 10 min upon NIR irradiation at 808 nm and 2 W/cm^2^ laser intensity.

The possibility to exploit the biocompatible and FDA-approved Prussian blue nanoparticles for bacteria and biofilm photothermal ablation has recently become a new research topic. Those nanoparticles strongly absorb in the range of 700–750 nm due to the metal-to-metal charge transfer between Fe^II^ and Fe^III^ through the cyanide bridge [37]. The photothermally induced death of Gram-positive and Gram-negative bacteria using the PVP-coated Prussian blue nanoparticles was one of the first experiments performed within the topic [73]. The photothermal effect was investigated at 810 nm and 980 nm under a 1 W·cm^−2^ irradiance. By choosing a proper nanoparticle concentration, the bacteria was selectively eliminated from the HeLa cells. In a later study, published in 2017, the photothermally active Prussian blue nanoparticles were grafted on a glass surface via a layer-by-layer approach [74]. The self-assembled monolayers demonstrated photothermally induced antibacterial activity against Gram-negative (*E. coli*) and Gram-positive (*S. aureus*) bacterial strains. In order to overcome the NIR laser limits and to irradiate larger areas, the nanocages were used to induce the photothermal sterilization upon solar light irradiation [75]. In a very recent article, published in 2019, physically cross-linked PVA hydrogel films, containing Prussian blue nanoparticles, displayed a pronounced photothermal effect (up to Δ*T* ≅ 78 °C) under low NIR laser intensities (0.3 W/cm^2^) [76]. The resulting local temperature increase was sufficient to eradicate ≈76% of *P. aeruginosa* bacteria and mitigate *P. aeruginosa* biofilm growth. Another recent study, also published in 2019, validated an exogenous antibacterial agent consisting of zinc-doped Prussian blue nanoparticles against the methicillin-resistant *S. aureus* in vitro and in a rat model for a cutaneous wound infection [77]. The authors demonstrated that the photothermally induced local heat, triggered by the irradiation at 808 nm and 1.2 W/cm^2^, accelerates the release and penetration of ions into bacteria, resulting in an alteration of the intracellular metabolic pathways and causing bacterial death without a systemic toxicity. In the latest study, published in 2020, thinly sprayed nanocomposite films containing Prussian blue nanoparticles displayed a pronounced effect against *P. aeruginosa* bacteria (84% bacterial death) and a moderate effect against *S. aureus* bacteria (69% bacterial death) under a low intensity (0.35 W/cm^2^) NIR irradiation [78].

Similar to the Prussian blue nanoparticles, CuS nanoparticles display a strong absorption in the NIR region, mainly within the 900–1200 nm range, with an efficient thermal relaxation resulting from the *d*–*d* energy band transition of the Cu^2+^ ions. The very low production costs together with a low cytotoxicity make the CuS nanoparticles a feasible alternative to the widely used gold nanoparticles for photothermally induced bacteria ablation [33,79].

**Figure 6:**
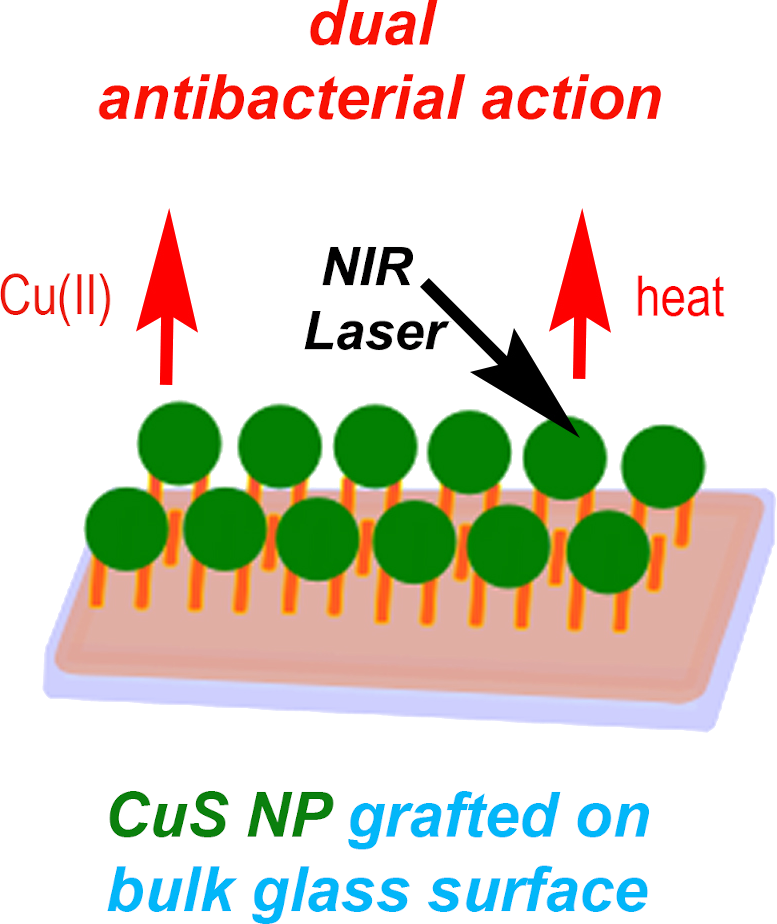
Dual antibacterial action of CuS nanoparticle monolayers on glass. Figure reprinted with permission from [85], copyright 2020, under the terms of the Creative Commons Attribution 4.0 International License, http://creativecommons.org/licenses/by/4.0/.

Interestingly, the early publications on CuS nanoparticles focused only on the antibacterial effect of the released Cu^2+^ ions but no mention was made in terms of their photothermally induced antibacterial activity [80–81]. Currently, there are still a few studies relating the application of the photothermal effect on CuS to eradicate bacteria. A multifunctional core/satellite nanostructure was engineered by decorating CuS nanoparticles onto the surface of a NaYF_4_:Mn/Yb/Er@photosensitizer doped with SiO_2_ This allowed for the integration of photodynamic and photothermic therapy to improve the treatment against multidrug-resistant bacteria [82]. It was demonstrated that such nanodevices exhibited superior antibacterial activity towards drug-resistant *S. aureus* and *E. coli*. In a recent study, poly(5-(2-ethyl acrylate)-4-methylthiazole-g-butyl)/copper sulfide nanoclusters were prepared to efficiently capture and eliminate levofloxacin-resistant Gram-negative and Gram-positive bacteria upon NIR laser irradiation [83]. The authors demonstrated that the conjugated nanoclusters significantly inhibited the levofloxacin-resistant bacteria after 5 min of 980 nm NIR laser exposure (1.5 W/cm^2^). In another recent publication, a dual-functional nanosystem based on ultrasmall CuS nanodots (≈6 nm) was developed to heal multidrug-resistant bacteria-infected chronic wounds [84]. The photothermal effect of such nanosystems (laser irradiation at 808 nm and 2.5 W/cm^2^) induced a strong in vitro and in vivo effect against drug-resistant pathogens, including methicillin-resistant *S. aureus* and extended-spectrum β-lactamase-resistant *E. coli*. Another study, published in 2020, involved the synthesis of CuS nanoparticle monolayers on glass [85]. As shown in Figure 6, the antibacterial activity of these monolayers is based on two different mechanisms: (i) slow and sustained copper release from the CuS NP-glass samples, (ii) local temperature increase caused by a photothermal effect under NIR laser irradiation.

Carbon nanotubes are another valuable class of nanomaterials. They have high photothermal efficiency under NIR irradiation which excites the longitudinal phonon resonance along the nanotube. The resonance peaks can be tuned by changing the tube length [86]. Therefore, carbon nanotubes also have potential to be used in the NIR-triggered photothermal bacterial treatment. It was shown that multiwall carbon nanotubes functionalized with antibodies against the group A *Streptococcus* bacteria were capable of photothermally ablating the planktonic and biofilm-residing bacteria upon 800 nm laser exposure (1.3 W/cm^2^) for 10–120 s [87]. The authors also found that, upon NIR irradiation, the antibody-labelled nanotubes displayed an enhanced antibacterial action on both planktonic cells and biofilms when compared to carboxylated multiwall carbon nanotubes. The use of carbon nanotubes functionalized with NIR-absorbing fluorophores was introduced in a work published in 2019 [88]. This nanohybrid platform was found to be an effective photothermal agent against bacteria. Temperatures as high as 92 °C were observed after 15 min of NIR laser irradiation (808 nm, 1 W/cm^2^), resulting in 77% planktonic *P. aeruginosa* cell death. In addition, polyurethane nanocomposites containing the same hybrid nanomaterials were also able to eliminate all the surface-grafted *P. aeruginosa* cells under NIR light irradiation.

Reduced graphene oxide, which is characterized by a broad absorption spectrum, is another well-known suitable agent for the NIR photothermal conversion [89]. This material is prepared by assembling polyelectrolyte-stabilized reduced graphene sheets on a quartz surface to efficiently generate localized heating under simulated solar light irradiation, resulting in a >90% eradication of airborne bacteria [90]. Magnetic reduced graphene oxide functionalized with glutaraldehyde was synthesized to capture and eradicate both Gram-positive (*S. aureus*) and Gram-negative (*E. coli*) strains under NIR light irradiation [91]. It was shown that a solution containing 80 ppm of the nanostructures provided rapid and efficient eradication of up to 99% of both Gram-positive and Gram-negative bacteria within 10 min of NIR laser irradiation. In a very recent publication, poly(vinyl alcohol) hydrogel incorporating reduced graphene oxide composites (MoS_2_/Ag_3_PO_4_) was fabricated to yield a highly efficient sterilization upon irradiation during wound healing [92]. The hybrid hydrogel exhibited excellent antibacterial properties against both *S. aureus* and *E. coli* under co-irradiation with 660 nm visible light and 808 nm near-infrared light for 10 min, due to the synergistic action of the photodynamic and the photothermal effect. The combination of the photothermal and photodynamic features of the graphene oxide nanoparticles with different sizes was also reported to eradicate Gram-positive and Gram-negative strains upon 630 nm light irradiation due to temperature increase and formation of ^1^O_2_ [93].

Functionalized iron oxide nanoparticles can also be used for photothermally induced bacteria eradication. It was demonstrated that the NIR-absorbing nanoparticles functionalized with recyclable iron oxide were capable of eliminating Gram-positive (*S. aureus*) and Gram-negative bacteria (*E. coli*) quickly and effectively [94]. To this end, iron oxide nanoparticles were coated with catechol-conjugated poly(vinylpyrrolidone) sulfobetaine and then self-assembled with poly(3,4-ethylenedioxythiophene). The latter polymer is capable of absorbing NIR light while capturing the bacteria, effectively releasing heat under NIR irradiation.

Among other examples of photothermally active nanoparticles applied for bacteria eradication it is worth mentioning the MoS_2_ nanoparticles. These nanoparticles have a good biocompatibility and a high photothermal conversion efficiency over a broad NIR range [95]. PEG-MoS_2_ nanoflowers with strong NIR absorption and peroxidase-like activity displayed in vitro fast antibacterial action on Gram-negative ampicilin-resistant *E. coli* and Gram-positive endospore-forming *B. subtilis* [96]. In another publication multifunctional chitosan-functionalized magnetic MoS_2_ nanoparticles were engineered to combat bacterial infections by integrating bacterial conjugation and NIR-triggered photothermal sterilization [97]. In a very recent publication, polyethylene glycol (PEG)-MoS_2_ nanosheets additionally functionalized with an antibody (anti-protein A lgG) and polydopamine were applied to induce NIR-photothermal ablation (>99.99%) of *S. aureus* both in biofilms and in infected tissues [98].

Molybdenum oxide nanoparticles display a strong absorption in the NIR region, originating from the intervalence charge-transfer transition between the Mo^5+^ and Mo^6+^ states [99]. Ag nanocubes supported on plasmonic MoO_3−_*_x_* nanosheets were designed to be a highly efficient NIR-driven antibacterial agent [100]. This approach enhanced the nanocube antibacterial activity towards *E. coli* and *S. aureus* upon NIR light irradiation due to the local temperature increase, release of silver ions and bacterial membrane oxidation.

Tungsten oxide nanocrystals also have photothermal properties due to strong absorption in the range of visible and NIR light [101]. Recently, it was demonstrated that WO_3−_*_x_* nanocrystals display an efficient antibacterial activity towards *E. coli* based on their capability to induce bacterial membrane stress, which is enhanced by the photothermal effect upon sunlight irradiation [102]. Antibacterial continuous flow poly(dimethylsiloxane)-based microreactors with microchannels were fabricated using catechol-grafted poly(*N*-vinylpyrrolidone) and NIR-active Cs_0.33_WO_3_ nanoparticles [103]. Upon NIR light activation (808 nm; 80 W/cm^2^), a pronounced photothermal antibacterial effect was observed during continuous operation for up to 30 days, in which a reduction of more than 99.9% of viable Gram-negative (*E. coli*) and Gram-positive (*S. aureus*) bacteria was observed. In another recent study, NIR-responsive cesium tungsten oxide (CsWO_3_) nanoparticles were immobilized into polymer dots and demonstrated a high antibacterial activity due to the generation of photothermal heat upon 5 min of NIR irradiation (laser intensity 2 W/cm^2^) [104].

Recent studies have been exploring strategies in which sunlight is used instead of NIR lasers to trigger the photothermal effect. For example, thiolated cobalt-doped ZnO nanoparticles were synthesized to photo-inhibit the efflux pump in multidrug-resistant bacteria [105]. The antibacterial activity of this nanosystem against methicillin-resistant *S. aureus* was found to be 100% at a 10 µg/mL concentration and 15 min exposure to sunlight. These authors also found the same antibacterial effect with thiolated iron-doped nanoceria upon sunlight exposure [106].

## Conclusion

Considerable progress in the field of antibacterial treatment has been made due to the exploitation of the photothermal properties of nanomaterials which can generate a fast, efficient and localized temperature increase to efficiently eradicate several bacterial strains (including those resistant to conventional agents), both in the planktonic and in the biofilm form. In addition, depending on the hybrid nature of these nanomaterials, the photothermal action can be synergistically coupled with an antibacterial ion release, antibiotic release or with photocatalytic reactions, leading to the generation of reactive oxygen species (i.e., photodynamic action). In this review we have briefly summarized the current state-of-the-art in the use of different types of photothermally active nanoparticles against both Gram-positive and Gram-negative bacterial strains. However, there are still critical questions to be addressed for a successful implementation of this approach. Firstly, the majority of the published studies are devoted to the use of expensive plasmonic gold nanoparticles, whose accumulation in the body is still controversial and may lead to several adverse effects. In this direction, nanoparticle toxicity must also be studied carefully. For example, a few studies demonstrated the low cytotoxicity of CuS nanoparticles on HDF human fibroblast cells [107] while others identified a viability decrease in the HUVEC and RAW 264.7 cells when the nanoparticle concentration used was higher than 100 µg/mL [108]. The apparent discrepancy in the obtained results might arise from a variation in the nature of nanoparticles, nanoparticle concentration, cell type or in the surface coating. Therefore, for comparison reasons, several independent experiments should be performed using the same type of nanomaterial. Nontoxic, inexpensive and highly efficient photothermal nanoparticles, such as the FDA-approved Prussian blue nanoparticles, should be explored more systematically. Secondly, given the imposed restrictions on the use of colloidal nanoparticles due to long-term stability and reusability issues, alternative approaches such as nanocomposite materials (e.g., gels, thin films, printed surfaces) should be studied more intensively. Thirdly, since the use of high-intensity lasers to achieve pronounced photothermal effects should be avoided, highly concentrated colloidal solutions need to be used which in turn disfavor their long-term stability and increase their preparation costs. In order to circumvent these issues, polymeric nanocomposite materials and printed surfaces were developed to enable the use of photothermal treatments in the clinical settings offering stable, reusable and protective surfaces which can be remotely activated by light on demand. In addition, by combining materials with static and photothermally induced antibacterial properties one can apply low-laser intensities and achieve significant results. For example, the gold and Prussian blue nanoparticles do not display static antibacterial action on their own; however, in combination with other materials, such as silver or copper sulfide, they acquire a dual effect as shown for the copper sulfide nanoparticle case. Another example is carbon nanotubes which can damage bacterial membranes without the application of NIR light exposure [109]. However, the nanoparticle stability should always be verified, especially in the case when several nanoparticles are combined.

Moreover, more studies demonstrating the advantages of using sunlight as opposed to lasers should be performed. Finally, since most of the used nanoparticles and hybrid nanomaterials absorb in the NIR bio-transparent window, this allows for the application of such materials in treating infections and eliminating biofilms that are formed at the surface of indwelling medical devices. Even though promising for clinical application, this approach needs to be intensively investigated through preclinical studies and clinical trials.
